# Development of Recombinant Antibodies and Its Application in Immunomagnetic Separation-Based Rapid Detection of *Vibrio cholerae* in Aquatic Environments

**DOI:** 10.4014/jmb.2405.05003

**Published:** 2024-10-01

**Authors:** Haiyang Zhang, Quan Wang, Haoxiang Zhuang, Qiu Lin, Wenchao Wang, Fangyu Ye, Saqib Nawaz, Jiangang Hu, Cuiqin Huang, Huifang Yin, Weidong Sun, Xiangan Han, Wei Jiang

**Affiliations:** 1 Shanghai Veterinary Research Institute, the Chinese Academy of Agricultural Sciences (CAAS), 518 Ziyue Road, Shanghai 200241, P.R. China; 2 Engineering Research Center for the Prevention and Control of Animal Original Zoonosis, Fujian Province, College of Life Science, Longyan University, Longyan, 364012, Fujian, P.R. China; 3 College of Veterinary Medicine, Nanjing Agricultural University, 210095, Nanjing, Jiangsu, P.R. China

**Keywords:** *Vibrio cholerae*, immunomagnetic separation, recombinant antibodies, sample pretreatment, rapid detection, immunomagnetic beads

## Abstract

Cholera caused by *Vibrio cholerae* remains a major public health concern in many countries. The greatest obstacle to detection of *V. cholerae* contamination in drinking water or aquatic environments mainly relates to sample preparation steps, especially the enrichment step. In this study, immunomagnetic separation methods were developed based on sequence-defined recombinant antibodies (rAbs) against *V. cholerae*, then used for the specific and efficient enrichment of *V. cholerae* in water samples. Using the variable region genes of the anti-*V. cholerae* monoclonal antibodies (mAbs) 5F2, the full-length IgG rAbs (R5F2) were produced using mammalian human embryonic kidney 293T cells. Two antibodies, 5F2 and R5F2, were used to prepare immunomagnetic beads (IMBs), and their capture efficiencies (CEs) were evaluated. The results showed that 0.4 mg of 5F2-IMBs and R5F2-IMBs exhibited good CEs (96.0% and 75.9%, respectively) against *V. cholerae* within 40 min. The IMBs could still effectively capture *V. cholerae* in large-volume reaction systems (5 ml to 25 ml). The CEs of 5F2-IMBs and R5F2-IMBs ranged from 90.2% to 70.7% and 65.1% to 44.2%, respectively. Furthermore, 5F2-IMBs and R5F2-IMBs did not show significant cross-reactivity with other bacteria and exhibited high specificity. When R5F2-IMS was used in combination with quantitative real-time PCR, the detection limit was approximately 5 colony-forming units/25 ml after enrichment for 4 h. Our results suggest that the rAbs produced herein could provide useful alternatives to traditional hybridoma-based antibodies for accurate detection of *V. cholerae* in food safety and environmental monitoring.

## Introduction

Cholera, caused by the *Vibrio cholerae* bacterium, is an acute infectious disease that has evolved into a significant global public health concern owing to the accumulation of fatalities across various regions [1, 2]. The World Health Organization estimates that *V. cholerae* infects 1-4 million people and claims up to 143,000 lives annually [3, 4]. *V. cholerae*, a gram-negative foodborne pathogen, is widely dispersed on chitinous surfaces in marine habitats globally [5]. *V. cholerae* infections can occur when a susceptible individual consume water contaminated with the bacterium from environmental sources such as lakes and rivers [6, 7]. In urban Dhaka, Bangladesh, 30% of source water samples collected from the households of cholera patients were positive for *V. cholerae* [8]. Although *V. cholerae* frequently results in moderate symptoms, (*e.g.*, vomiting and dehydration), it can be fatal if not treated in time [9]. Inadequate aquaculture management may has been identified as a key contributor to disease outbreaks, causing significant economic losses [10]. Aquatic products, integral to the human diet, may harbor various pathogenic bacteria, including *V. cholerae* [11]. Early diagnosis and confirmation of *V. cholerae* infections are prerequisites for the rapid implementation of control measures.

For the existing rapid detection methods, sample pretreatment is an important preliminary step, especially for target molecules present at low concentrations. Rapid enrichment of target molecules not only removes impurities that can potentially affect subsequent detection but also concentrates sample solutions, enhancing detection sensitivity and streamlining the entire process [12]. Although environmental waters are important reservoirs of *V. cholerae* detecting the bacterium in these samples is often challenging because of their presence in low levels and the interference from other bacteria. Therefore, rapid selective enrichment of the bacterial population from water samples is necessary, followed by selective isolation, and culture in a selective medium is generally required. Magnetic beads (MBs) with properties such as strong magnetism and superparamagnetic effects have been extensively used for biological detection, biosensors, in vitro diagnostics, and biochips to speed up the detection process and increase its sensitivity and specificity [13-15]. Cells and proteins can be enriched using MBs, which can be combined with active substances (e.g., antigens, antibodies, and receptors) to bind to target molecules [16]. Immunomagnetic separation (IMS) using MBs coated with specific antibodies to produce immunomagnetic beads (IMBs) is widely used to capture target pathogens from complex food matrices and background microflora through antibody-antigen interactions [12, 17-19]. With the advantages of being rapid, sensitive, simple, and cost-effective, IMS has been extensively employed to enrich various pathogens such as *Escherichia coli* [20], *Vibrio parahaemolyticus* [21, 22], *Salmonella* [23], *Avian influenza virus* [24] and *SARS-CoV-2* [25]. IMS technology can detect target pathogens in a single tube, which is primarily determined by the antibodies involved. Traditional antibodies, including monoclonal antibodies (mAbs) and polyclonal antibodies, are generally used to produce IMBs using IMS [26, 27]. Briefly, IMS is used to enrich pathogens during sample preparation, and quantitative real-time PCR is used to detect target DNA through amplification. However, challenges such as insufficient inter-batch stability and long preparation cycles associated with mAbs, ethical concerns, and the high costs of animal-dependent production led to the exploration of recombinant antibodies (rAbs) [28, 29].

The development of molecular biology techniques has enabled us to identify the mAbs sequence and express the mAbs in mammalian expression systems [30], which can reduce the dependence on hybridomas and animals [31, 32]. More importantly, the mAb DNA fragments in hybridoma cell lines may undergo mutations and loss over multiple generations [29]. In contrast, the expression of rAbs in stable sequences can avoid the challenges associated with repeated freeze-thaw cycles, and minimises the risk of loss of antigenicity, alleviates the financial burden associated with hybridoma cell preservation and maintenance [28, 33]. In addition, rAbs can reduce batch-to-batch variation of antibody preparation [34]. Consequently, rAbs can be industrially produced and efficiently purified, reaching large-scale production and significantly reducing costs [35, 36]. RAbs can be conjugated to captured bacteria on the surface of MBs and have been widely used for the treatment and diagnosis of multiple diseases, including cancer and non-cancer diseases [37-40]. Recently, rAbs have been increasingly used for the sensitive detection of pathogens, including *Pseudomonas aeruginosa* [41] and *Staphylococcus aureus* [42]. However, the application of rAbs in immunoassays has not been widely developed.

To the best of our knowledge, this is the first study to use rAbs for IMS, an important method for promoting rAb applications, opening a novel pathway for rapid diagnostic screening and field analysis. We employed the variable regions (VR) of hybridomas that secrete high-affinity and specific antibodies against *V. cholerae*. The corresponding full-length IgG rAbs were constructed using a mammalian cell expression system incorporating both heavy and light chains. Furthermore, rAbs with enhanced specificity and sensitive were used to develop a reliable IMS to capture *V. cholerae* in water samples. The performance of rAbs-IMBs was characterized and compared with that of mAbs-IMBs. When rAbs-IMBs were used in combination with qPCR for *V. cholerae* detection, the newly developed R5F2-IMS-qPCR assay enabled the detection of this pathogen at low levels (5 CFU/25 ml) in spiked water samples within 4 h.

## Materials and Methods

### Bacterial Strains and Culture Conditions

Four strains of *V. cholerae* were used: one clinical isolate (C0903) was used for subsequent optimization and other experiments, one isolate from seawater (W1608), and two isolates from seafood (F0553 and F0374). In addition, another four *Vibrio* species and other bacterial strains were used in the present study (Table 1). The *Vibrio* strains were cultured with fresh 3% NaCl LB broth medium and incubated overnight at 37°C. The other bacterial strains were grown on fresh LB medium at 37°C with shaking at 180 revolutions per minute (rpm) for 12 h. To obtain viable bacterial counts, 10-fold serial dilutions of overnight cultures were made in phosphate-buffered saline (PBS, pH 7.2), and 100 μl of the dilutions were plated on the nutrient agar.

### mAb and rAb Production

A concentration of inactivated *V. cholerae* was adjusted to 10^8^ CFU/ml, and a volume of 0.5 ml was mixed with an equivalent amount of complete Freund's adjuvant (Sigma-Aldrich, USA). The prepared antigens were injected subcutaneously into 6- to 8-week-old female BALB/c mice. Booster antigens were prepared by mixing inactivated *V. cholerae* with an equal amount of incomplete Freund's adjuvant and were injected every 2 weeks. After four immunizations, serum antibody titers in mice were detected using an enzyme-linked immunosorbent assay (ELISA). Mice with high serum titers underwent impulse immunization, with 10^5^ CFU multiple *V. cholerae* in 100 μl PBS, injected intraperitoneally. The specific anti-*V. cholerae* mAbs were prepared using immunized spleens and fused with S/P20 cells. Hybridoma cells were screened in HAT medium (Solarbio, China), and monoclonal cells were obtained after four rounds of subcloning. MAbs were prepared (5F2) against ascites from the selected cell lines for future use. The mAb subtypes were determined using mAb subtype identification kits (Southern Biotech, USA).

To express R5F2 in mammalian human embryonic kidney (HEK) 293T cells in vitro, the total RNA of 5F2 cells was extracted using Trizol. Subsequently, cDNA was synthesized with SuperScript III Reverse Transcriptase (Invitrogen, USA) according to the manufacturer’s instructions using the following primers (Eurofins Genomics): IgG1-RT, 5'-TTATTTACCAGGAGAGTGGGA-3'; Ig kappa-RT, 5'-CTAACACTCATTCCTGTTGA-3'. The VR sequences were amplified using degenerate primers with High-Fidelity DNA Polymerases (Ex Taq, Takara). PCR cycling conditions were as follows: 95°C for 2 min, followed by 30 cycles at 98°C for 10s, 58°C for 30s, 72°C for 60s, and a final elongation step at 72°C for 8min. The segments of 5F2 VR were cloned into chimeric expression plasmids pcDNA3.1 (+) for R5F2 production in 293T cells with the TransIT-X2 Dynamic Delivery System (Mirusbio, USA). The R5F2 was purified using a Protein G affinity chromatography column (Cytiva, USA). Twenty column volumes of the binding buffer (20 mM sodium phosphate buffer, pH 7.0) were used to prewash and equilibrate the resin. The supernatant was immediately placed on a column. A binding buffer was used to wash off the nonspecific binding. The specific antibodies were eluted from the column using 0.1 M glycine buffer at pH 2.7 and immediately neutralized by a one-fifth volume of 1 M Tris-HCl solution (pH 9.0). Purified antibodies were evaluated using sodium dodecyl sulfate-polyacrylamide gel electrophoresis (reducing SDS-PAGE). All animal experiments were approved by the Animal Ethics Committee of the Shanghai Veterinary Research Institute, Chinese Academy of Agricultural Sciences (No. SYXK<HU>2020-0027).

### Analysis of 5F2 and R5F2 Binding Activity by ELISA

The purified antibodies were analyzed using ELISA, which tests the antigen-binding ability of the antibodies. Inactivated *V. cholerae* (10^8^ CFU/well) were coated onto a micro-ELISA plate (Nunc, USA) at 4°C overnight. The wells were blocked with 200 μl of 1 % gelatin as described above and then washed three times with PBST, 100 μl of the same concentration of purified 5F2 and R5F2 (100 μl) at different dilution concentrations was added and incubated for 1.5 h at 37°C. The wells were washed three times before adding 100 μl of horseradish peroxidase (HRP)-conjugated anti-mouse IgG (1:10,000 dilution in PBST; Sigma-Aldrich) and incubated at 37°C for 50 min. The wells were washed three times, the ELISA was completed with TMB-ELISA Substrate Solution (Tiangen, China) and 2 M H_2_SO_4_, and the absorbance at 450 nm was monitored using a microplate reader (Corning, China). Samples reaching an *OD_450_* value ≥ 0.3 (mean *OD* + *3SD*) were considered positive, with 0.3 set as the assay cut-off value. The specificity against *V. cholerae* antibodies was tested for the selection of other bacteria (Table 1) using ELISA.

### Preparation of 5F2-IMBs and R5F2-IMBs

Carboxyl MBs of different sizes (180 nm, 750 nm, 1 μm, 2 μm, and 5 μm) were purchased from Shanghai Aolun Micro Materials Co., Ltd. (China). Before use, the MBs (10 mg/ml) were mixed, 100 μl MBs were transferred to an EP tube, and the MBs were separated using a magnetic particle concentrator (Beaver, China). The MBs were carefully rinsed three times with a 0.025 M 2-(N-morpholino) ethane sulfonic solution containing 0.05% Tween-20 (MEST, pH 6.0) after the supernatant was carefully removed. The beads were thoroughly mixed, resuspended in 400 μl MES (0.025 M, pH 6.0) containing 5 mg/ml of 1-ethyl-3-(3-dimethylaminopropyl) carbodiimide hydrochloride and 5 mg/ml N-hydroxy succinimide, and incubated at 37°C for 30 min. After being washed three times with PBST (0.01 M PBS containing 0.05% Tween 20, pH 7.4), the activated IMBs were mixed with anti-*V. cholerae* antibodies, followed by a 1 h incubation at 37°C with gentle shaking. Finally, the IMBs were resuspended in 2 mg/ml concentration in PBST and stored at 4°C until use.

After being washed three times with MEST (MES containing 0.05% Tween 20, pH 7.0), the activated IMBs were mixed with 60 μg of anti-*V. cholerae* antibodies (5F2 or R5F2) and diluted in 500 μL PBST (0.01 M PBS containing 0.05% Tween 20, pH 7.4). The mixture was incubated on a rotator at 25°C with a 25 rpm rotation (Hula Mixer; Life Technologies). After the IMBs were placed on the magnetic particle concentrator and washed with PBST, 1 ml of blocking solution (0.01 M PBST, pH 7.4; 1% BSA) was added with gentle shaking for at least 30 min. Finally, the IMBs were resuspended in 2 mg/ml in stock solution (0.01 M PBST, pH 7.4; 0.02% NaN_3_, 0.5% BSA) and stored at 4°C until further use.

### Capture of *V. cholerae* Using 5F2-IMBs and R5F2-IMBs

*V. cholerae* was grown in 3% NaCl LB medium at 37°C until the stationary phase was reached (*OD_600_* =1). Serial 10-fold dilutions of *V. cholerae* at different concentrations were prepared in PBST. In separate experiments, a mixture of 5F2-IMBs, R5F2-IMBs, and bacteria was incubated on a rotator (25 rpm) at room temperature and separated using a magnetic particle concentrator. Negative controls were prepared without 5F2 and R5F2. The tubes were carefully placed in a magnetic concentrator to isolate the IMB-bacteria complexes, and the supernatant was then removed. The 5F2-IMBs and R5F2-IMBs with bacterial complexes and supernatant were cultured on 3%NaCl LB agar plates at 37C for 24 h for colony counting. The capture efficiency (CE) of the 5F2-IMBs and R5F2-IMBs was calculated using the following equation: CE (%) = C_0_ / (C_0_+C_a_) × 100%, where C_0_ is the total number of *V. cholerae* present in the sample (colony-forming unit (CFU)/ml) and Ca is the number (CFU/ml) of cells not bound to 5F2-IMBs and R5F2-IMBs.

### Optimization of IMS based on 5F2 and R5F2

For method optimization, 5F2-IMBs and R5F2-IMBs were compared. Specific comparisons of the CEs obtained using five different concentrations of 5F2-IMBs and R5F2-IMBs (0.1, 0.2, 0.3, 0.4, and 0.5 mg), six incubation times (10, 20, 30, 40, 50, and 60 min), and different concentrations of *V. cholerae* suspensions (ranging from 10^1^ to 10^5^ CFU/ml) were analyzed. Under the optimal conditions, the CEs for different capture systems (5, 10, and 25 ml) of *V. cholerae* were tested with five amounts of IMBs (0.4, 0.8, 1.2, 1.6, and 2.0 mg) in PBST.

### Specificity and Stability of 5F2-IMBs and R5F2-IMBs

To evaluate the specificity of the 5F2-IMBs and R5F2-IMBs methods, we used four strains of *V. cholerae* (C0903, F0553, F0374, and W1608), four *Vibrio* spp. strains (*V. alginolyticus*, *V. mimicus*, *V. vulnificus*, and *V. parahaemolyticus*), and four other foodborne bacteria (*Escherichia coli*, *Listeria monocytogenes*, *Salmonella Typhimurium*, and *Enterobacter sakazakii*). 5F2-IMBs and R5F2-IMBs capture and plating were performed as described above, and CEs were calculated. The stability of 5F2-IMBs and R5F2-IMBs was tested by storing IMBs for five durations (0, 3, 6, 9, and 12 months) at 4°C, followed by re-examination of CEs and performance with non-*V. cholerae* pathogens. Coefficients of variation (CV) were obtained for each parameter from four independent measurements.

### Analysis of Spiked Aquaculture Water Samples Using 5F2-IMS-qPCR and R5F2-IMS-qPCR

The effectiveness of the established 5F2-IMBs and R5F2-IMBs methods in aquaculture water was evaluated using locally collected samples of aquaculture water. All samples were negative for the target bacteria when tested using standard culturing methods. There were no samples that tested positive for *V. cholerae*. This study used the optimized 1-, 5-, 10-, and 25-ml capture volumes of *V. cholerae* to compare the CEs of different aquaculture water-simulated samples. The utility of 5F2-IMBs and R5F2-IMBs as surveillance tools for the detection of *V. cholerae* in water samples was also evaluated. 5F2-IMBs and R5F2-IMBs were used in combination with qPCR (Primers in Table 2) to detect *V. cholerae*, and the newly developed 5F2-IMS-qPCR and R5F2-IMS-qPCR assays were used to investigate aquaculture water samples containing low quantities of *V. cholerae*. Aquaculture water (25 ml) was spiked with serially diluted *V. cholerae* to achieve final inoculation concentrations of 5.0 × 10^0^ CFU/25 ml and 5.0 × 10^1^ CFU/25 ml. Blank controls were prepared using non-spiked samples. Each sample was then placed in a shake flask containing 225 ml of liquid 3% NaCl LB medium. Following incubation at 37°C for varying periods (0, 1, 2, 3, 4, 5, or 6 h), 25 ml of spiked aquaculture water or enrichment solution was collected and mixed with R5F2-IMBs to capture *V. cholerae* under optimal conditions. After IMS, the concentrated cells were plated, and bacterial counts were determined. Concurrently, the IMB-*V. cholerae* complexes were resuspended in 100 μl of deionized water. The bacterial suspension was then boiled at 100°C for 10 min to release genomic DNA, which was used as the template for qPCR to quickly and efficiently confirm the presence of *V. cholerae* in positive samples.

## Results

### Production of mAbs of 5F2 and R5F2

**Fig. 1:**
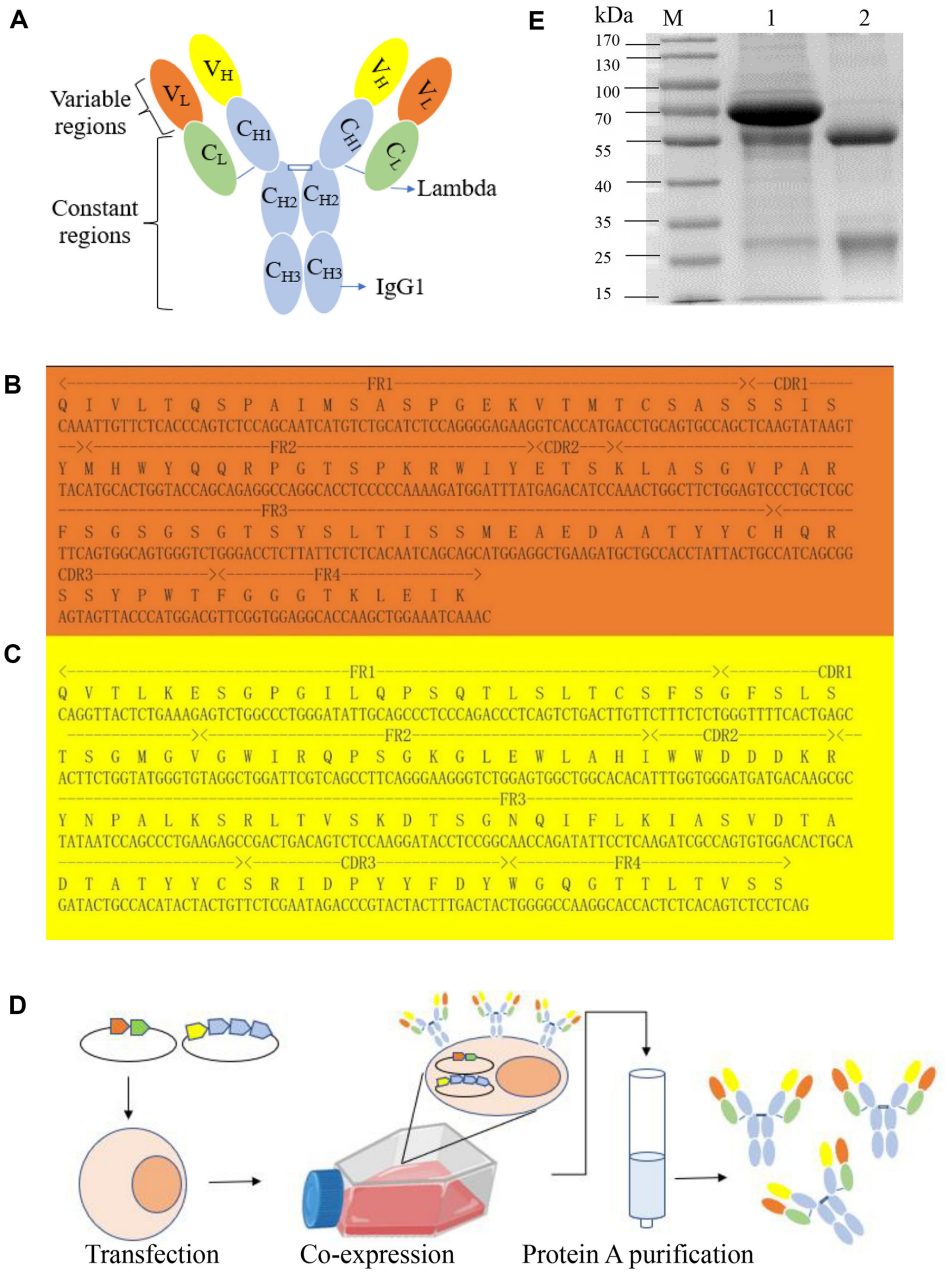
Scheme of the production of full-length rAbs. (**A**) Schematic diagram of antibody mode, (**B**) Light chain variable region sequence; (**C**) Heavy chain variable region sequence; (**D**) Representation of HEK293T-based recombinant anti- *V. cholerae* antibody generation; (**E**) SDS-PAGE of the eukaryotic-expressed antibody supernatant and the purified rAbs. RAbs supernatant culture (Lane 1) and purified RAbs (Lane 2).

The 5F2 subtype was identified as IgG1 heavy chain (HC) and lambda light chain (LC) (Fig. 1A). Total RNA from hybridoma cells was extracted and reverse transcribed into cDNA. The VR series gene sequences of the antibodies were PCR-amplified and sequenced. The sequences of the LC (Fig. 1B) and HC (Fig. 1C) of 5F2 were successfully obtained. Based on the VR of 5F2, the HC and LC segments of R5F2 were subcloned into the eukaryotic expression plasmid, pcDNA3.1 (+) (Fig. 1D). Following the purification of the cell supernatant using Protein A beads, the expression and purity of R5F2 were determined using reducing SDS-PAGE (Fig. 1E) used to verify the size of the two bands of the antibody chains. The molecular weights of VL and VH were approximately 25 kDa and 50 kDa, respectively. These values align with the theoretical expectations, providing preliminary evidence for successful expression of the antibody.

### Analysis of R5F2 and 5F2 Binding Characteristics Using ELISA

**Fig. 2:**
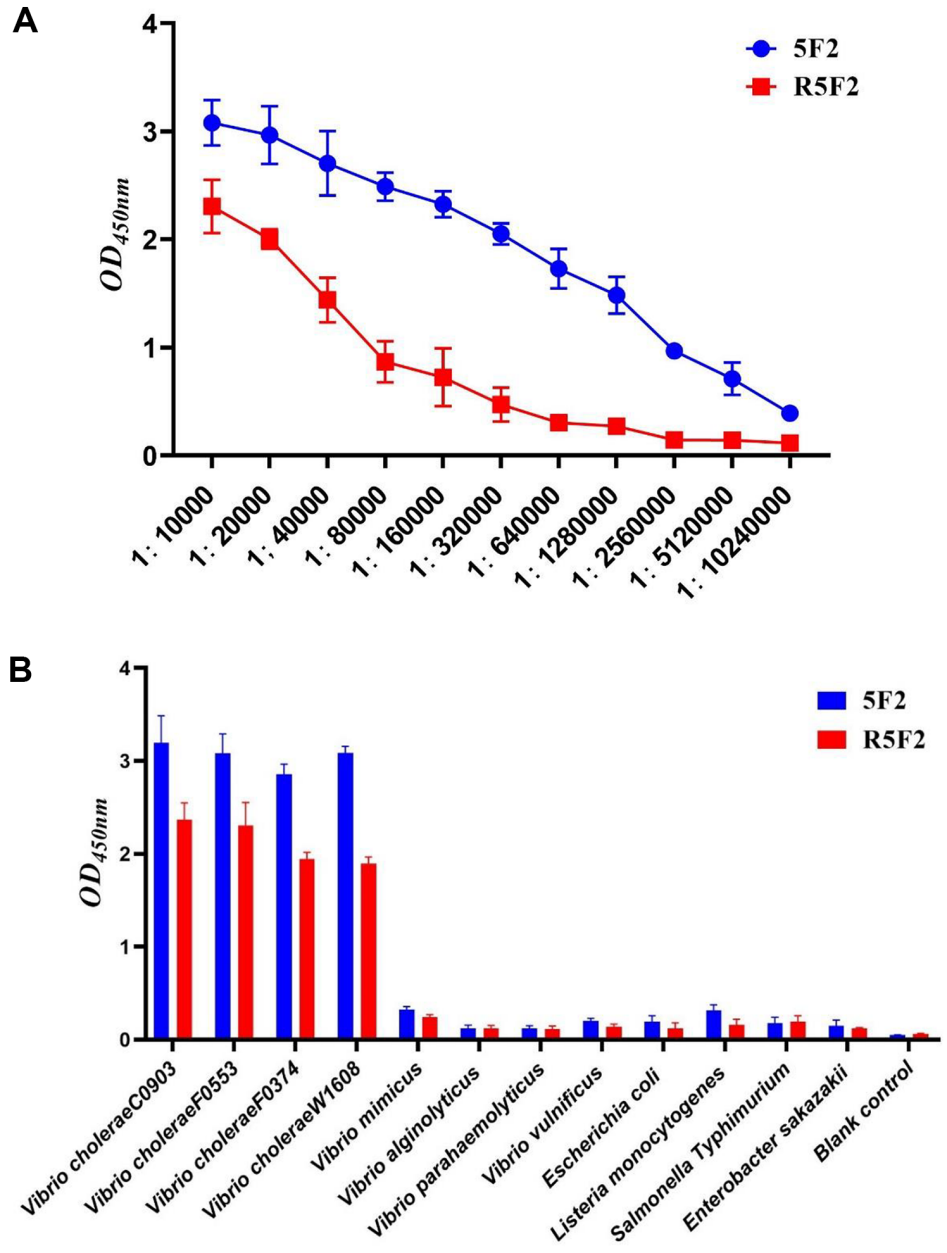
Determination of 5F2 and R5F2 binding ability to *V. cholerae* by ELISA. (**A**) ELISA of anti-*V. cholerae* antibody pair with 10^8^ CFU/ml of *V. cholerae*; (**B**) Specificity of the ELISA method. In the ELISA, incubation with *V. cholerae* alone gave a strong signal, while the signals for other bacteria were weak and similar to those of the negative control.

The antibody titers of purified 5F2 and R5F2 were detected using indirect ELISA. The 5F2 titer was 1:2,560,000, whereas the R5F2 titer was as high as 1:160,000 (Fig. 2A). The specificity of 5F2 and R5F2 and their binding properties to various bacteria were measured using indirect ELISA. Both antibodies strongly reacted with the four strains of *V. cholerae*. However, no reaction was observed for the other eight non-*V. cholerae* bacteria (Fig. 2B). These results indicate that 5F2 and R5F2 can detect *V. cholerae* with high sensitivity and specificity.

### Preparation of 5F2-IMBs and R5F2-IMBs

**Fig. 3:**
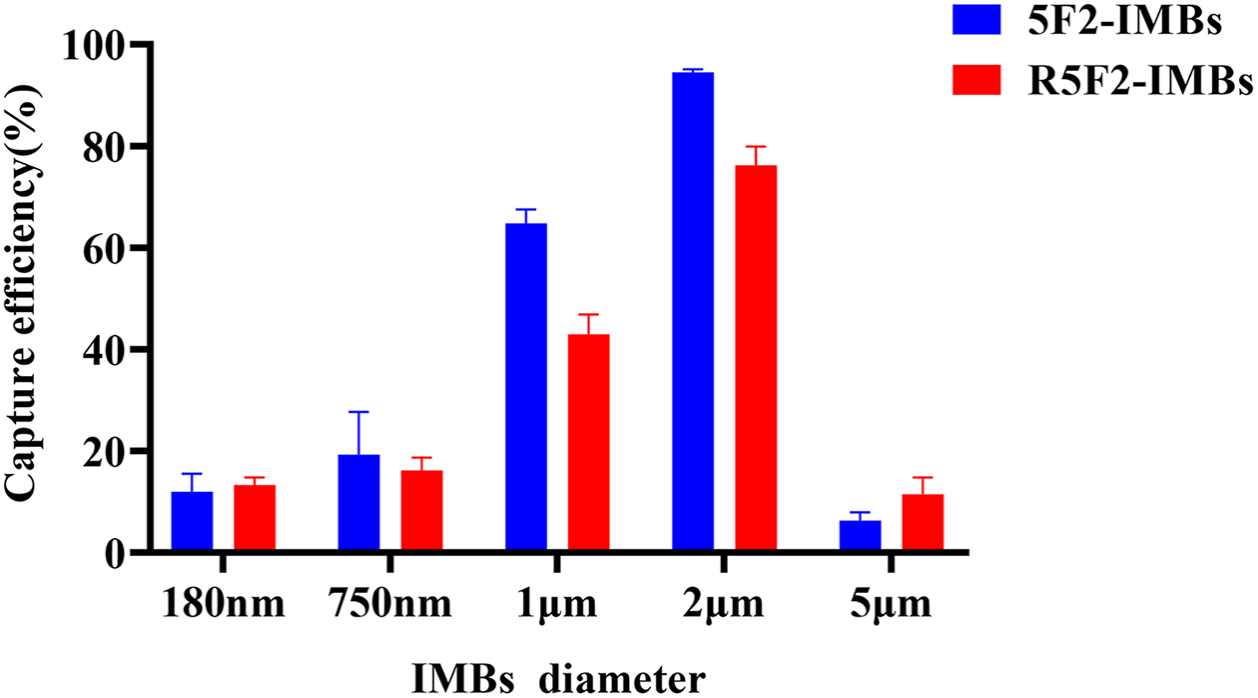
Effects of IMBs diameters. The capture efficiencies (CEs) were determined by using 10^3^ CFU/ml of *V. cholerae* *C0903* in PBST within 40 min.

To assess the influence of MB size on the CEs, different beads sizes (180 nm, 750 nm, 1 μm, 2 μm, and 5 μm) were compared at the same concentration of *V. cholerae* (10^3^ CFU/ml). The results showed that 2 μm-diameter IMBs showed the highest CEs for both 5F2-IMBs (94.5%) and R5F2-IMBs (76.2%). The CEs of 1 μm IMBs were slightly lower than those of 2 μm IMBs (65.8% for 5F2-IMBs and 43.0% for R5F2-IMBs), but much higher than those of other IMBs with diameters of 180 nm, 750 nm, and 5 μm (<19.3% for 5F2-IMBs and <16.2% for R5F2-IMBs)(Fig. 3).

### Optimization of 5F2-IMBs and R5F2-IMB

**Fig. 4:**
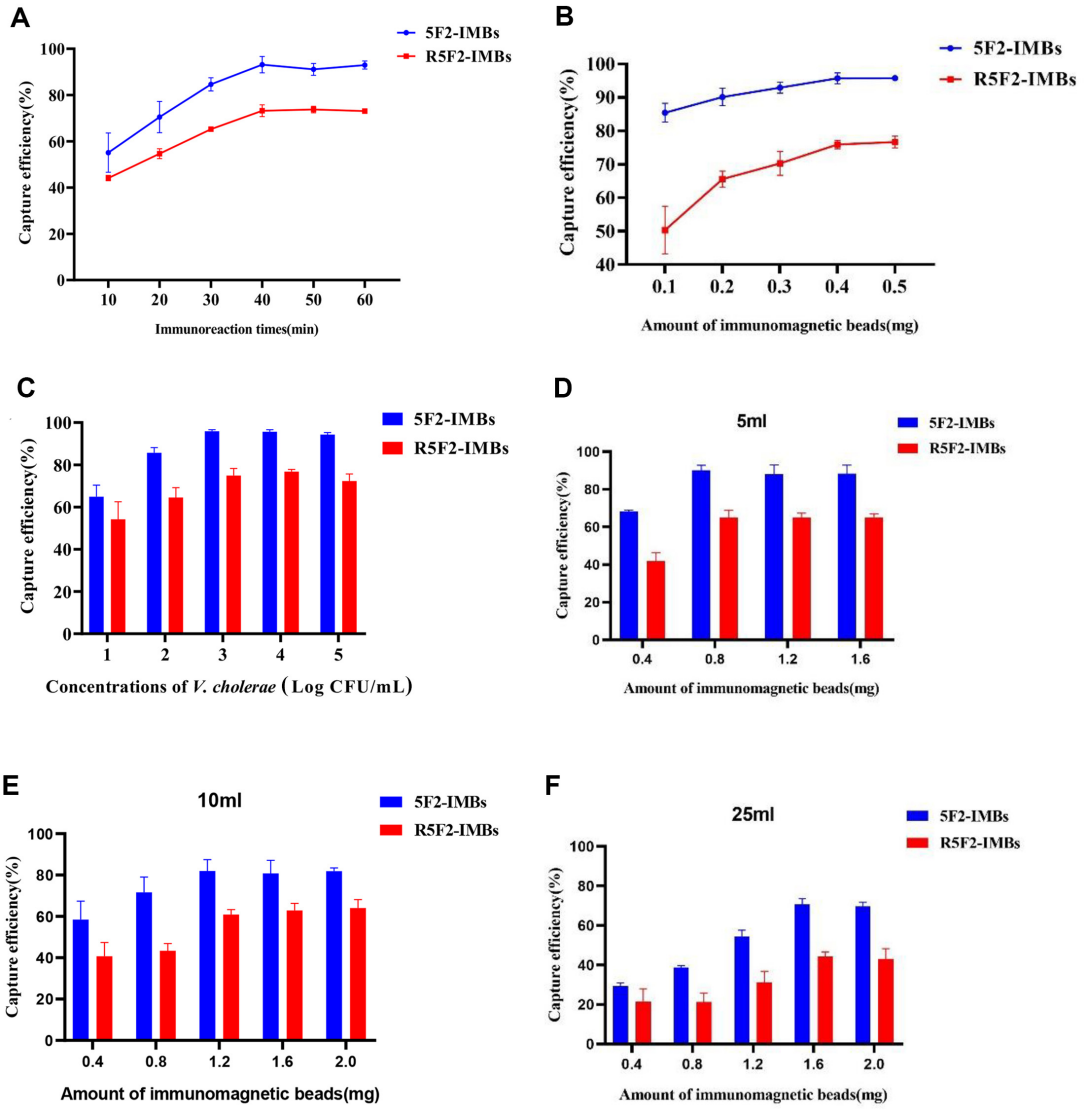
Optimization of the 5F2-IMBs and R5F2-IMBs. Effects of Immunoreaction time (**A**), amounts of IMBs (**B**), and bacterial concentration (**C**) on the capture efficiencies (CEs) of *V. cholerae* *C0903*. Optimized amounts of IMBs in 5 ml (**D**), 10 ml (**E**) and 25 ml (**F**) systems on the CEs of *V. cholerae* *C0903*. The CEs were determined by using 10^3^ CFU/ml of *V. cholerae* *C0903* in (**A**), (**B**), (**D**), (**E**) and (**F**); 0.4 mg of IMBs in (**A**) and (**C**); and a 40 min reaction time in (**B**), (**C**), (**D**), (**E**), and (**F**).

Within the first 40 min of incubation, the CEs of 5F2-IMBs rapidly increased from 55.1% (10 min) to 93.2%(40 min), and the CEs of R5F2-IMBs rapidly increased from 44.1% (10 min) to 73.2% (40 min). Incubation times longer than 40 min did not show any further significant increase in CEs, indicating that a 40 min incubation was sufficient to achieve the maximum enrichment efficiency (Fig. 4A). Additionally, the optimal amount of IMBs required for CEs was explored. When the number of IMBs was increased from 0.1 to 0.5 mg, the CEs of 5F2-IMBs increased from 85.4% to 95.7% and those of R5F2-IMBs increased from 50.3% to 75.9%. The CEs of both 5F2-IMBs and R5F2-IMBs exhibited stability when the amount of IMBs was greater than 0.4 mg (Fig. 4B), demonstrating that 0.4 mg of IMBs was sufficient for the enrichment of *V. cholerae* in a 1 ml reaction system. These results confirm that 5F2-IMBs and R5F2-IMBs reached their optimal CEs under the same conditions. As shown in Fig. 4C, when the bacterial concentration ranged from 10^1^ to 10^5^ CFU/ml, the CEs of 5F2-IMBs and R5F2-IMBs gradually increased, peaking at 95.9% and 75.5%, respectively, indicating that 10^3^ CFU/ml was the optimal capture concentration for *V. cholerae*. 5F2-IMBs and R5F2-IMBs could capture more than 64.9% and 54.2%, respectively, of *V. cholerae* at a low bacterial concentration of 10 CFU/ml, indicating the excellent performance of the IMBs produced in this study.

The CEs of 5F2-IMBs and R5F2-IMBs for *V. cholerae* under optimal conditions at different capture volumes (5, 10, and 25 ml) are shown in Fig. 4D–4F (concentration of *V. cholerae*: 10^3^ CFU, reaction time: 40 min). In 5 ml PBST, the CEs of 5F2-IMBs and R5F2-IMBs reached their maximum values (90.2% and 65.1%, respectively) when the amount of IMBs was 0.8 mg. In 10 ml PBST, the CEs of 5F2-IMBs and R5F2-IMBs reached their maximum values (82.0% and 61.6%, respectively) with 1.2 mg of IMBs. In 25 ml PBST, the CEs of 5F2-IMBs and R5F2-IMBs reached their maximum values (70.7% and 44.2%, respectively) with 1.6 mg of IMBs. These results demonstrated that the CE of IMBs conjugated with either 5F2 or R5F2 gradually decreased as the volume of the reaction system increased. However, even in the largest system (25 ml), the CE of 5F2-IMBs could still reach more than 70%, and the CEs of R5F2-IMBs could reach more than 40%.

### Specificity and Stability of 5F2-IMBs and R5F2-IMBs

Eight non-cholera *Vibrio* spp. strains were used to assess the specificity of 5F2-IMBs and R5F2-IMBs. The results revealed that the CEs of 5F2-IMBs was 24% for *V. mimicus* and < 5.3% for other bacteria (Table 1). Similarly, R5F2-IMBs demonstrated good specificity with CEs < 8.5% for non-cholera *Vibrio* spp. and showed a minimal non-specific reaction to *V. mimicus* (CEs 18.7%).

After storage at 4°C for 12 months, the CEs of the 5F2-IMBs and R5F2-IMBs were evaluated. The results indicated that the mean CEs of the 5F2-IMBs and R5F2-IMBs were as high as 95.0% and 75.7%, respectively, which was comparable to the CEs of freshly produced IMBs (Table 3). This implies that the 5F2-IMBs and R5F2-IMBs reagents maintain their efficacy for at least 12 months at 4°C without affecting the capture performance. Repetitive experiments with different storage periods demonstrated a CV of less than 5% (Table 3), affirming the favorable reproducibility of the established method.

### Sample Analysis and Comparison of 5F2-IMS-qPCR and R5F2-IMS-qPCR

**Fig. 5:**
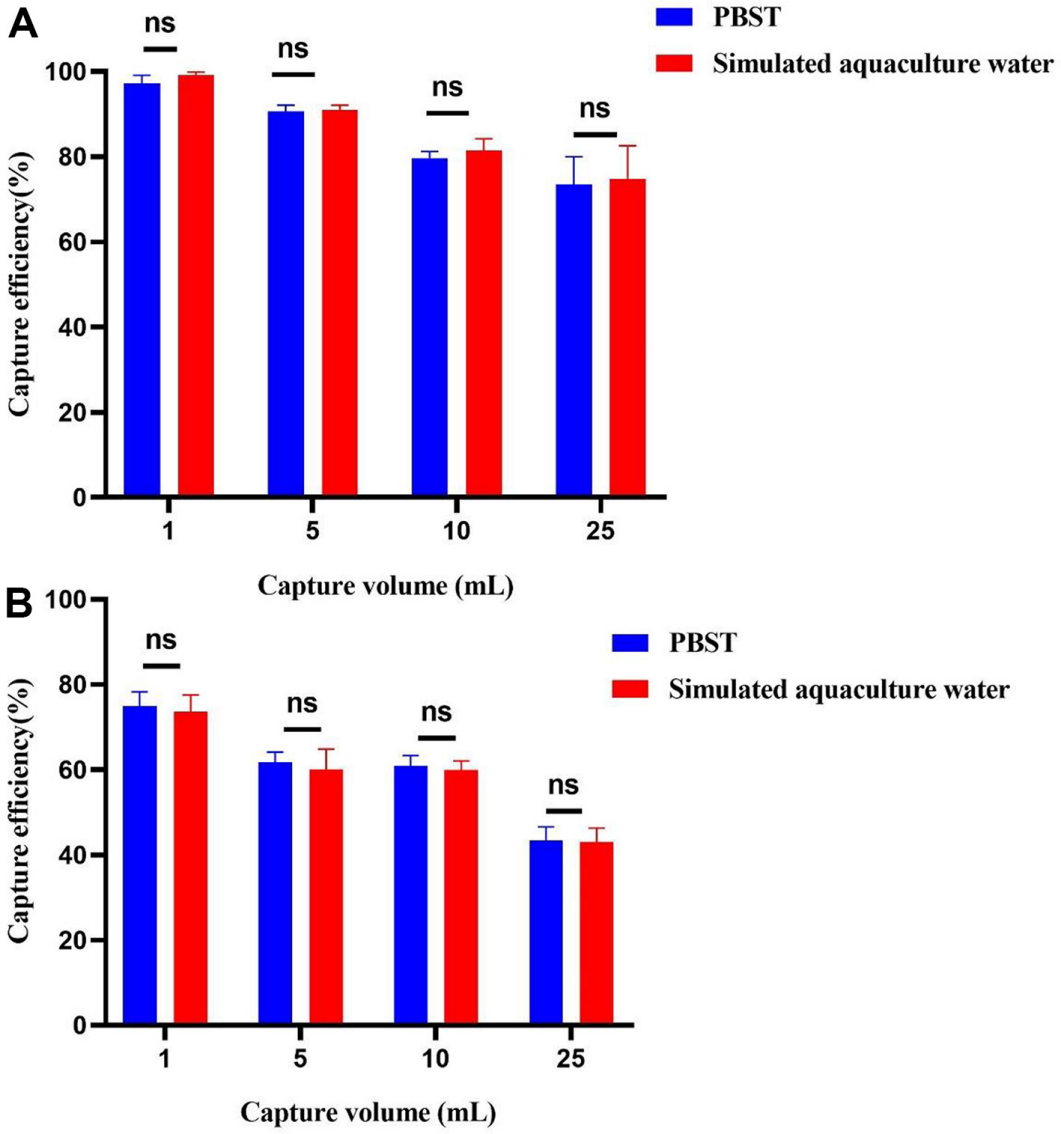
Results of *V. cholerae* in simulated samples. (**A**) 5F2-IMBs detection of *V. cholerae* *C0903* in simulated aquaculture water samples; (**B**) R5F2-IMBs detection of *V. cholerae* *C0903* in simulated aquaculture water samples. Different capture volumes of 1 ml, 5 ml, 10 ml, and 25 ml adjusted the capture efficiencies (CEs) of *V. cholerae* *C0903* for different simulated aquaculture water samples. The capture efficiency was determined by using 10^3^ CFU/ml of *V. cholerae* *C0903* (a 40 min reaction time). (*ns*: *p* > 0.05)

The difference in *V. cholerae* CEs between 5F2-IMBs and R5F2-IMBs in the PBST and aquaculture water samples was not significant (*p* > 0.05), even with different capture systems (1, 5, 10, and 25 ml) (Fig. 5A and 5B). The detection limits of the 5F2-IMS-qPCR and R5F2-IMS-qPCR assays for *V. cholerae* without enrichment were 2×10^4^ CFU/25 ml and 5 × 10^4^ CFU/25 ml, respectively. When the number of *V. cholerae* in water samples was below the limit of detection for qPCR amplification, an enrichment step was required. In this study, for water samples inoculated with 5 CFU/25 ml of *V. cholerae*, both the 5F2-IMS-qPCR and R5F2-IMS-qPCR methods required 4 h of enrichment to obtain a positive result (Table 4).

## Discussion

The limitations associated with the traditional use of monoclonal and polyclonal antibodies, as well as concerns about the unnecessary use of animals for antibody production, have also prompted people to explore RAbs for food safety applications [32]. Hybridoma cells were established based on *V. cholerae*. The genetic materials of mAbs can be extracted from hybridoma cell lines and reproduced serially through appropriate expression systems. Compared to *E. coli* and *Pichia pastoris*, mammalian cells are more capable of introducing protein folding, post-translational modifications, and then folding into appropriate protein structures and showing high affinity by providing dimer binding sites similar to those of natural antibodies [43-45]. Therefore, 293T cells were used to express the recombinant antibody (R5F2) in this study. Natural signal peptides from mammalian cells are often replaced with well-known heterologous signal peptides to improve the yield of rAbs [46, 47]. This is because the use of natural signal peptides derived from humans can achieve higher efficiency than the use of signal peptides from various species, or even natural immunoglobulin G signal peptides. For this study, we chose the IL-2 signal peptide, which is consistently effective at increasing protein secretion in vitro and is widely used in both commercial protein production and gene therapy research [48, 49]. Uncontrollable HC and LC ratios affect the quality of mAbs by causing aggregate formation and glycosylation modification [50]. To improve the level of secretion of recombinant proteins, the optimal ratio of HC and LC plasmids encoding 5F2 transfected into 293 T cells was 4:5. This ratio enables the yields of R5F2 to reach 3.5 mg per 10 mL cell culture. Transiently transfected HEK293cells only achieve low mAbs expression levels of tens to hundreds of milligrams per liter during batch culture [51]. This result is consistent with previous research findings, as there are differences in the manufacturability, affinity, and efficacy of antibodies produced in HEK cells compared to those produced by stably transfected CHO cells [52]. As part of downstream program and product quality assurance, other vector designs and host cell lines can be used to increase antibody yields [53].

MBs of different diameters were conjugated with 5F2 and R5F2 to prepare the 5F2-IMBs and R5F2-IMBs, respectively, for capturing *V. cholerae*. Large specific surface areas of MBs (*e.g.*, 180–1,000 nm in diameter) can significantly enhance the efficiency of isolating molecular targets from the environment, attributed to the rapid binding kinetics of smaller IMBs to target cells [54-56]. We found that the 2 μm and 1 μm IMBs had CEs higher than those of nanoscale IMBs and the 5 μm beads (all below 13%). Our finding was consistent with previous reports that larger beads were capable of traveling through a larger volume of sample solution with a higher chance of capturing target bacteria, compared to smaller beads containing less magnetic material. However, the CEs decreased when the IMB size reached 3-5 μm [57, 58]. Furthermore, despite being conjugated with MBs of different diameters, 5F2-IMBs and R5F2-IMBs showed similar trends in their CEs of *V. cholerae*.

Apart from the diameter of the MBs, the CEs of the IMS method also depends on the dose of antibodies conjugated to the IMBs, immunoreaction time, and amount of IMBs [12, 59]. When 60 μg of 5F2 or R5F2 was conjugated to 2 μm MBs, both the 5F2-IMBs and R5F2-IMBs exhibited maximum CEs. Increasing the dose of antibodies above 60 μg did not improve the CEs of IMBs, which indicated that 60 μg of 5F2 or R5F2 was sufficient to conjugate MBs and had a good capture rate performance (data not shown). To streamline the capture process in drinking water, aquaculture water, or seawater without the need for preliminary procedures such as centrifugation, a series of reaction systems of 5–25 ml was tested. Notably, the CEs of 5F2-IMBs and R5F2-IMBs were 85.7% and 64.6%, respectively, in the 1 ml test system containing a *V. cholerae* concentration of 10^2^ CFU/ml. Although the reaction system was expanded to 10 ml with a *V. cholerae* concentration of 10^2^ CFU/ml, the CEs could still reach 82.0% for 5F2-IMBs and 61.6% for R5F2-IMBs. Expanding the capture system may increase the sensitivity of subsequent detection techniques, enabling a more comprehensive and accurate identification of *V. cholerae*. These results indicate the potential benefits of expanding the capture system. However, the results also showed that the CEs of R5F2-IMBs were lower than that of 5F2-IMBs. Further improvements can be used to increase the expression level and binding activity of recombinant antibodies. In addition, the existing antibody sequences can be analyzed and modified with computational technology, which may improve antibody function and enhance antibody performance [60, 61].

When the established 5F2-IMBs and R5F2-IMBs were used to capture for non- *V. cholerae* strains, a little cross-reactivity was observed with the *V. mimicus* strains that the CEs of the 5F2-IMBs and R5F2-IMBs for *V. mimicus* strains were 24% and 18.7%, respectively, which may be attributed to significant genomic similarities between *V. cholerae* and *V. mimicus* [62]. Cross-reactivity depends not only on a particular antibody, but also on the complexity of a sample and the rarity of the target protein [63]. With the advent of whole genome sequencing and advances in bioinformatics, it is now possible to mine the sequences of potential V.cholerae-specific target genes using various algorithms [64]. Meanwhile computational methods can help to make progress in computer simulation design of antibodies, such as antibody structure modelling and antibody-antigen complex prediction [65]. Subsequent studies of the rAbs in this study could also be optimised based on target proteins to improve their specificity. But fortunately, even if cross-reacting bacterial species were bound by IMBs, *V. cholerae* cells could be specifically detected by coupling the 5F2-IMBs and R5F2-IMBs with a downstream method such as qPCR. Our results demonstrated that 5F2-IMBs and R5F2-IMBs could effectively differentiate and enrich *V. cholerae* from other common *Vibrio* spp. strains and foodborne bacteria, indicating that 5F2-IMBs and R5F2-IMBs had good specificity.

No significant difference in the CEs was observed, indicating that aquaculture water does not affect the efficiency of 5F2-IMBs and R5F2-IMBs (Fig. 5). These results suggested that complex background materials, such as feed, excretions, and various chemicals in aquaculture water, did not interfere with the interaction between the target cells and IMBs, indicating that both 5F2-IMBs and R5F2-IMBs have good pathogen-binding capacity. To date, the reference methods used to detect *Vibrio* in water systems have relied mainly on microbiological techniques via enrichment steps and isolation in culture media. It can take many days to detect growth, making rapid decisions impossible, and can be biased toward other *Vibrio* spp. [66]. IMBs are useful in sample preparation to enrich low numbers of target bacteria and eliminate interfering substances in aquaculture water. Thus, they improve the detection limit and allow for the rapid and sensitive detection of *V. cholerae*. Various molecular DNA-based detection methods have been developed for *V. cholerae*, and the detection limits vary widely from one method to another [67, 68]. In the present study, we decreased the overall analysis time using a combination of IMS and qPCR. Our results suggest that the 5F2-IMBs and R5F2-IMBs produced herein can be used to specifically concentrate *V. cholerae* in aquaculture water within 40 min. The DNA of *V. cholerae* captured by the IMBs was released by heating the IMB- *V. cholerae* complexes in a boiling water bath within 5 min, and the genetic material of *V. cholerae* was detected by qPCR within 1 h. Thus, the newly developed 5F2-IMS-qPCR and R5F2-IMS-qPCR assays can be used to detect low numbers of *V. cholerae* in large volumes of aqueous samples within half a workday, whereas the conventional culture-based method requires several days.

## Conclusion

This study demonstrated the effectiveness and practicability of an IMS method based on rAbs. rAb-IMBs demonstrated comparable performance to mAb-IMBs, exhibiting efficient and rapid enrichment of *V. cholerae* in aquaculture water. The rapid detection of low numbers of bacteria is crucial to prevent water or aquatic products from being contaminated by *V. cholerae*. The newly developed R5F2-IMS-qPCR assay effectively combines sample preparation and detection into a single platform, enabling analysis to be completed within half a workday. In summary, this study confirmed that rAbs can be used to produce IMBs to capture *V. cholerae*, which can be used for rapid enrichment of *V. cholerae* in water samples in emergencies, especially in areas with limited resources.

## Figures and Tables

**Table 1:** Bacterial strains and the specificity performance of the IMS system.

Species	Source, strain (No.)	CE^b^	
		5F2-IMBs	R5F2-IMBs
*V. cholerae*	C0903	96.0%	75.9%
*V. cholerae*	F0553	94.3%	74.2%
*V. cholerae*	F0374	92.7%	71.6%
*V. cholerae*	W1608	93.0%	72.1%
*V. mimicus*	Ref^a^. ATCC 33653	24.0%	18.7%
*V. alginolyticus*	Ref. ATCC 17749	4.4%	8.3%
*V. parahaemolyticus*	Ref. ATCC 33847	4.8%	8.5%
*V. vulnificus*	Ref. ATCC 27562	5.3%	8.4%
*Escherichia coli*	Ref. ATCC 35150	2.2%	3.9%
*Listeria monocytogenes*	Ref. ATCC 21635	1.8%	5.7%
*Salmonella Typhimurium*	Ref. ATCC 14028	1.5%	4.5%
*Enterobacter sakazakii*	Ref. ATCC 21550	2.3%	3.2%

^a^Ref., reference strains; ATCC, American Type Culture Collection; ^b^CE, capture efficiency.

**Table 2:** Primers and probe used in the study.

Assay	Primer/probe name	Sequence (5'-3')
qPCR	ompW -probe:	CGCGGGTATTGCCTCGGTAGTACCT
	RT-VC-ompW-F:	GCTACCAAGAAGGTGACTTTATTG
	RT-VC-ompW-R:	AACTGCCAACTCACTTTGAGTGTTT

**Table 3:** Results of inter-assay variance analysis for storage time.

IMS	Storage time	Capture Efficiency (CE)					Standard deviation	Coefficient of variation
	Month	1	2	3	4	Mean	(SD) %	(CV) %
5F2- IMBs	0	95.2%	94.2%	97.4%	94.8%	95.4%	1.39	1.46
	3	93.2%	95.0%	93.9%	96.5%	94.6%	1.43	1.51
	6	94.9%	97.1%	97.6%	94.8%	96.1%	1.42	1.48
	9	93.0%	93.8%	97.6%	96.8%	95.3%	2.25	2.36
	12	94.8%	92.9%	95.2%	97.2%	95.0%	1.76	1.85
R5F2- IMBs	0	77.4%	77.5%	70.4%	74.5%	75.0%	3.34	4.45
	3	76.6%	74.9%	77.2%	75.5%	76.1%	1.01	1.33
	6	73.6%	72.5%	70.5%	75.6%	73.0%	2.13	2.92
	9	78.2%	74.2%	77.6%	75.3%	76.3%	1.89	2.48
	12	77.6%	74.8%	77.4%	72.8%	75.7%	2.26	2.99

**Table 4:** 5F2-IMS-qPCR and R5F2-IMS-qPCR method for the detection of *V. cholerae* in samples.

*V. cholerae* concentration (CFU/25 ml)	Enrichment time (h)	Method	
		5F2-IMS-qPCR	R5F2-IMS-qPCR
5 × 10^1^	0	−	−
	1	−	−
	2	−	−
	4	+	+
	6	+	+
	8	+	+
5 × 10^0^	0	−	−
	1	−	−
	2	−	−
	4	+	+
	6	+	+
	8	+	+
